# Rapid chondrolysis of the medial knee compartment after arthroscopic meniscal resection: a case report

**DOI:** 10.1186/s13256-016-0841-7

**Published:** 2016-04-01

**Authors:** Sylvain Steinmetz, François Bonnomet, Michel Rahme, Philippe Adam, Matthieu Ehlinger

**Affiliations:** Department of Orthopaedic and Trauma Surgery, Hôpitaux Universitaires de Strasbourg, 1, avenue Molière, 67098 Strasbourg cedex, France

**Keywords:** Arthroscopy, Case report, Complication, Knee, Rapid chondrolysis

## Abstract

**Background:**

Rapidly destructive osteoarthritis of the hip and rapid chondrolysis of the lateral compartment of the knee or the shoulder are rare, but have been previously described in the medical literature. To the best of our knowledge, no case of medial femorotibial compartment chondrolysis after arthroscopy has yet been described. We therefore submit the first case report.

**Case presentation:**

A 64-year-old white European man presented with right knee pain due to a medial meniscal tear with no other abnormality found on examination or imaging. An arthroscopic partial medial meniscectomy was performed and early evolution was favorable with no signs of infection. He developed knee pain 2 months later. X-rays showed a thinning of the medial compartment which was confirmed by computed tomography arthrogram. There was no articular effusion, mobility was conserved (0/0/125°), there was no laxity, and pain was localized to the medial femorotibial compartment, with no meniscal signs. There was a 8° varus deviation (versus 3° for his uninjured left knee). His blood work was normal. As there were no signs of infection, no aspiration was performed. Viscosupplementation was offered but refused by the patient. He is now waiting for a partial knee replacement.

**Conclusions:**

To the best of our knowledge, this is the first description of such a case. Rapid chondrolysis has been described in the hip, shoulder, and the lateral compartment of the knee. Infiltration of bupivacaine and lateral meniscectomy are the most frequently sited offending procedures. Concerning the medial compartment, cases of avascular necrosis have been reported after meniscectomy or use of radiofrequency devices. This case underlines the necessity of a thorough physical examination and complete radiological work up before any surgery. It must also drive us to use caution regarding meniscectomy, especially in patients over 60 years of age, and reminds us that patients must be informed of this potential complication.

## Background

Rapidly destructive osteoarthritis of the hip and rapid lateral femorotibial or glenohumeral chondrolysis are rare but known pathologies, and evolution is often unfavorable. Conservative treatment is complicated and largely ineffective, and arthroplasty is often required.

Although rapid chondrolysis is often idiopathic, in some cases particular events or risk factors can be identified. Some authors have incriminated intra-articular bupivacaine, pain pumps in the shoulder [1] or the knee [2], or the use of radiofrequency [3]. For the knee, an arthroscopic lateral meniscus resection has been found to be a risk factor, without a clear mechanism identified [4]. Finally, some authors have described acute chondrolysis of the knee involving accidental use of intra-articular chlorhexidine [5] or due to the presence of intra-articular foreign bodies [6–8].

To the best of our knowledge, no other case of rapid chondrolysis has ever been described in the medial femorotibial compartment after arthroscopic medial meniscectomy, notably in a case where no pain pump was utilized.

## Case presentation

**Fig. 1:**
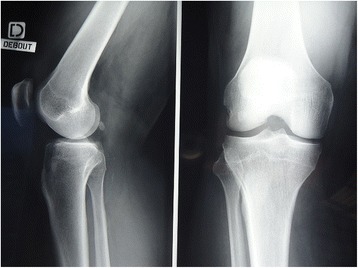
Preoperative radiography. Anterior-posterior and lateral view. No sign of arthritis on the medial compartment

**Fig. 2:**
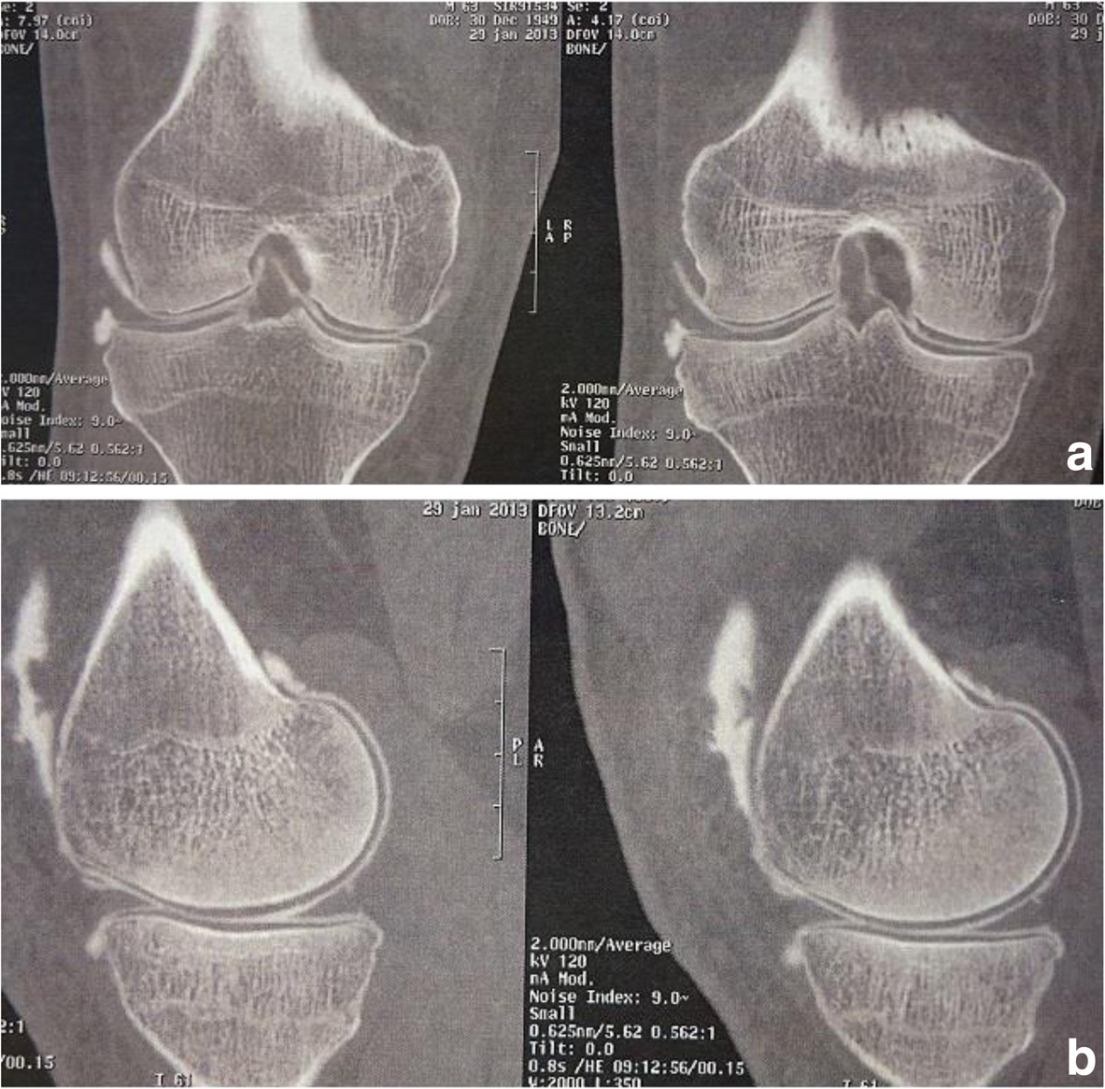
Preoperative arthrotomodensitometry, 6 weeks before arthroscopy. **a** Frontal view: no sign of chondrolysis. **b** Sagittal view: no sign of chondrolysis, only a little subchondral condensation. No sign of meniscal tear

**Fig. 3:**
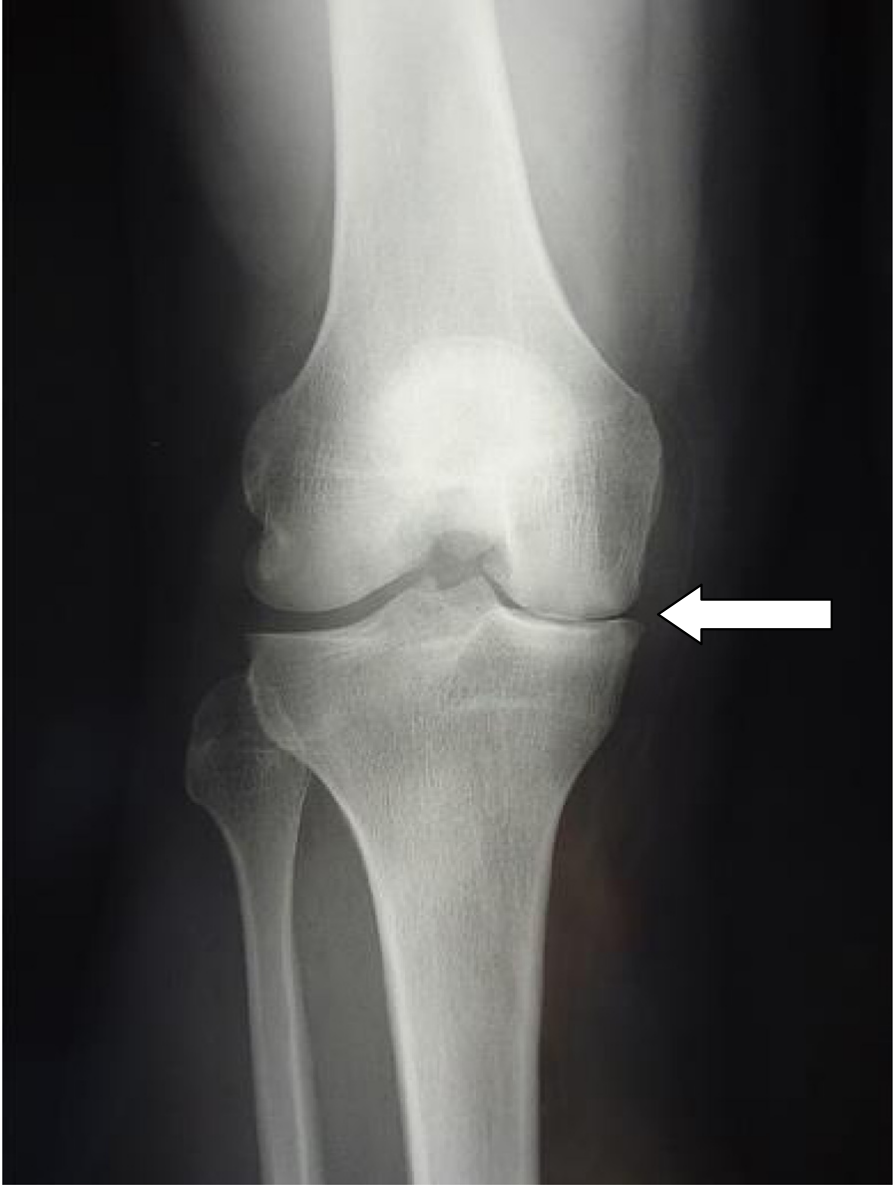
Postoperative radiography at 3 month follow-up. Anteriorposterior view, lateral view is not available. Complete medial femorotibial thinning (arrow)

**Fig. 4:**
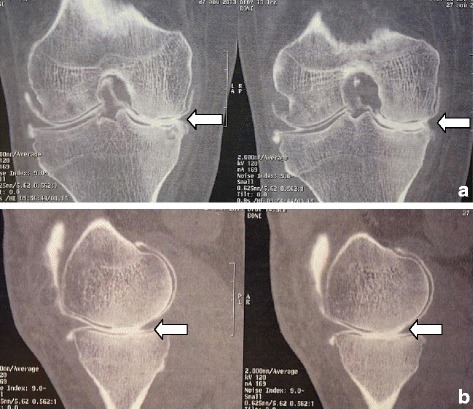
Postoperative arthrotomodensitometry at 4 month follow-up. **a** Frontal view: complete medial chondrolysis (arrow), with intraosseous arthritic cyst. **b** Sagittal view: complete chondrolysis (arrow)

A 64-year-old white European man presented with right knee pain due to an isolated medial meniscal tear of non-traumatic origin, confirmed on X-ray and computed tomography (CT) arthrogram (Figs. 1 and 2). An arthroscopic partial medial meniscectomy was performed and in the same session a grade 1a International Cartilage Repair Society (ICRS) cartilaginous lesion was revealed. His early postoperative course was favorable, without fever or other signs of infection. Unfortunately, his knee pain reappeared 2 months postoperatively and became invalidating within the following month, leading to another set of X-rays (anterior-posterior and lateral) that showed complete medial femorotibial joint thinning. A clinical examination showed neither effusion nor redness; he had a good range of motion (0/0/125°), no laxity, a slight varus morphology and anterior medial femorotibial pain with no meniscal pain. The X-rays were completed by a schuss view that confirmed an Ahlbäck stage III joint thinning (Fig. 3) and a CT arthrogram showed complete chondrolysis of his medial femorotibial compartment (Fig. 4). Standing long leg films showed an 8° varus deviation compared to 3° on his left knee. All blood work was normal, and no rheumatologic cause was identified. No genetic testing was performed. As there was no argument for infection, no aspiration was performed. Infiltration and viscosupplementation were proposed but refused by the patient. He is now waiting for a unicompartmental knee replacement.

## Discussion

Chondrolysis is the disappearance of articular cartilage due to lysis of the chondral matrix and the chondrocytes. It results in progressive thinning of the joint line, which can cause pain and swelling. Cases have been observed primarily in the shoulder [1] and the knee [4], but also have been described in the ankle [9]. To the best of our knowledge no case has been previously described in the literature regarding the medial compartment of the knee after arthroscopic meniscectomy without intra-articular pain pump.

Primary chondrolysis has been described in one case report affecting a woman in the third decade of her life (20 to 29 years) where an autoimmune cause was probable [10]. However, the mechanism of chondrolysis often remains unknown, and in these cases the articular damage is often rapid and occurs in young patients.

Secondary forms have also been described, with chemical or physical factors identified that have probably contributed to the chondrolysis. Some authors have incriminated intra-articular bupivacaine pain pumps, with the first cases seen in the shoulder [1, 11] but since described in the knee [2] and the ankle [9]. Noyes *et al*. [2] report 21 cases of rapid chondrolysis in the knee after using intra-articular pain pumps, with six cases of global knee damage, ten bicompartmental lesions and five unicompartmental lesions. They concluded their study by contraindicating the use of these pain pumps. Chu *et al*. [12] and Gomoll *et al*. [13] demonstrated on animal models that bupivacaine is cellulotoxic to chondrocytes. However, Gomoll *et al*. [14] moderated their conclusions in a second study; after evaluating the long-term effects of intra-articular bupivacaine, they observed an increase in cellular metabolism actually suggesting a healing process. Recently Braun *et al*. [15] showed that 0.5 % bupivacaine mixed with epinephrine induced a significant increase in cellular death of synoviocytes and that 0.5 % bupivacaine alone induced cellular lesions but mostly liberated metalloproteinase, suggesting an indirect harmful effect on the chondrocytes due to synoviocyte damage.

Antiseptic products can also be harmful to cartilage. Acute chondrolysis of the knee involving accidental use of intra-articular chlorhexidine has been described [5]. Recently, von Keudell *et al*. [16] showed that application of diluted povidone-iodine, independent of the concentration, was toxic to superficial chondrocytes if the contact lasted longer than 1 minute.

The use of radiofrequency or thermocoagulation was considered by some authors to be a risk factor for rapid chondrolysis of the glenohumeral joint after debridement or capsulorrhaphy [3], as well as in the hip after thermal excision of the labrum [17]. No such case has been described in the knee after using radiofrequency or thermocoagulation, but some cases of secondary avascular necrosis have been published [18, 19].

Some particular situations such as intra-articular loose bodies can cause chondral lesions, but usually have limited damage. Gliatis *et al*. [6] published a case of chondral injury due to migration of a Mitek RapidLoc meniscal repair implant. Sonnery-Cottet *et al*. [8] reported a case of tibial plateau chondrolysis after meniscal repair with a hybrid suture anchor. Jung *et al*. [7] reported a case of meniscal tear and chondral damage of the lateral compartment due to migration of a loose piece of cement after a medial unicondylar knee arthroplasty. The articular damage caused by a loose cement body differs based on the site; in the hip, it was responsible for a rapid and total chondrolysis requiring total arthroplasty [20].

In the knee, the most frequently described mechanical risk factor is arthroscopic lateral meniscus resection [4]. While the mechanism remains unknown, several theories have been postulated, including local mechanical overload due to changes in weight-bearing post-meniscectomy, or due to early and intense return to sports [4]. The lateral compartment is more often affected because the load distribution is more frequently modified following lateral meniscectomy [21], and because the lateral meniscus absorbs 70 % of the lateral load whereas the medial meniscus absorbs only 50 % [22]. After a meniscectomy, there is sagittal, frontal and rotational articular instability [23, 24] that can go unnoticed on physical examination [25], but it can, according to some authors [4], favor chondrolysis, especially if it is associated with intense physical activity. In our case, we hypothesize that there is medial damage due to excessive pressure post-meniscectomy causing probable articular damage.

Bojescul *et al*. [9] reported cases of rapid chondrolysis in the setting of articular laxity, as well as after the use of postoperative intra-articular bupivacaine. Mariani *et al*. [4] proposed a mechanical explanation with a localized articular overload and a residual laxity. The most plausible hypothesis is that these cases of rapid chondrolysis are actually multifactorial (Table 1).

**Table 1:** Review table of knee chondrolysis causes by different authors

Etiology	
Antiseptic products (chlorhexidine) [5]	
Radiofrequency or thermocoagulation [18]	
Intra-articular loose bodies [6]	
Arthroscopic lateral meniscus resection [4]	
Intra-articular bupivacaine pain pumps [2]	
Antiseptic products (povidone-iodine) [16]	
Autoimmune [10]	

There is no specific treatment for rapid chondrolysis and conservative solutions are largely ineffective, as the surgeon is essentially confronted with secondary osteoarthritis. Initial management consists of rest, non-weight bearing until the effusion has regressed, and symptomatic treatment of associated pain. Arthroscopic lavage and microfractures have not been shown to be useful [11]. Cartilage restoration procedures, anti-inflammatory medication, steroid injection and viscosupplementation can be proposed, but have equivocal results [9]. Use of hyaluronic acid could theoretically be effective because it suppresses fibronectin fragment-mediated chondrolysis, facilitates restoration of matrix components, and enhances the release of stromelysin-1 [26]. In a case of complete chondrolysis the only option seems to be arthroplasty. While prevention is difficult, the only way to avoid this rare but severe complication is to perform arthroscopies only in patients that have a clear lesion, and to avoid diagnostic arthroscopies. Therefore preoperative imagery needs to be thorough and precise.

This case underlines the need for a thorough radiological and clinical examination, the results of which must be documented prior to surgery. It also warns us against performing meniscectomy in patients over 60 years of age. Unfortunately, often the only available solution after complete chondrolysis is an arthroplasty (as is the case in the hip or shoulder).

## Conclusions

Knee arthroscopy is a frequently performed surgery, but one that carries certain risks. Chondrolysis of the lateral compartment is rare but has been described in the literature after lateral meniscectomy. Chondrolysis of the medial compartment has never been described to the best of our knowledge. The case we report should alert us to this potential complication, and its presence should be explained to the patient. It seems that while prevention is difficult, a thorough preoperative screening and confirmation of a validated surgical indication should help minimize risk.

## Consent

Written informed consent was obtained from the patient for publication of this case report and any accompanying images. A copy of the written consent is available for review by the Editor-in-Chief of this journal.
